# Study of Mosquito Aerodynamics for Imitation as a Small Robot and Flight in a Low-Density Environment

**DOI:** 10.3390/mi12050511

**Published:** 2021-05-02

**Authors:** Balbir Singh, Noorfaizal Yidris, Adi Azriff Basri, Raghuvir Pai, Kamarul Arifin Ahmad

**Affiliations:** 1 Department of Aerospace Engineering, Faculty of Engineering, Universiti Putra Malaysia, Serdang 43400, Malaysia; nyidris@upm.edu.my (N.Y.); adiazriff@upm.edu.my (A.A.B.); 2 Department of Aeronautical and Automobile Engineering, Manipal Institute of Technology, Manipal Academy of Higher Education, Manipal 576104, India; 3 Department of Mechanical and Manufacturing Engineering, Manipal Institute of Technology, Manipal Academy of Higher Education, Manipal 576104, India; raghuvir.pai@manipal.edu; 4 Aerospace Malaysia Research Centre, Faculty of Engineering, Universiti Putra Malaysia, Serdang 43400, Malaysia

**Keywords:** flapping frequency, stroke amplitude, wake capture, wing flexibility, rotational drag, low-density

## Abstract

In terms of their flight and unusual aerodynamic characteristics, mosquitoes have become a new insect of interest. Despite transmitting the most significant infectious diseases globally, mosquitoes are still among the great flyers. Depending on their size, they typically beat at a high flapping frequency in the range of 600 to 800 Hz. Flapping also lets them conceal their presence, flirt, and help them remain aloft. Their long, slender wings navigate between the most anterior and posterior wing positions through a stroke amplitude about 40 to 45°, way different from their natural counterparts (>120°). Most insects use leading-edge vortex for lift, but mosquitoes have additional aerodynamic characteristics: rotational drag, wake capture reinforcement of the trailing-edge vortex, and added mass effect. A comprehensive look at the use of these three mechanisms needs to be undertaken—the pros and cons of high-frequency, low-stroke angles, operating far beyond the normal kinematic boundary compared to other insects, and the impact on the design improvements of miniature drones and for flight in low-density atmospheres such as Mars. This paper systematically reviews these unique unsteady aerodynamic characteristics of mosquito flight, responding to the potential questions from some of these discoveries as per the existing literature. This paper also reviews state-of-the-art insect-inspired robots that are close in design to mosquitoes. The findings suggest that mosquito-based small robots can be an excellent choice for flight in a low-density environment such as Mars.

## 1. Introduction

**Figure 1:**
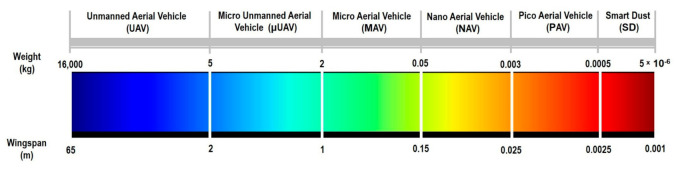
Spectrum-based drone size scale. Reproduced with permission from ref. [1]. Copyright 2017, Elsevier (Amsterdam, The Netherlands).

The role of robotic systems, including miniature unmanned autonomous ones over the years, has expanded considerably. With the rapid advancement in sensor and robotic technologies, these robotic vehicles are envisaged to be assigned various tasks, including vector disease control, atmospheric analysis, disaster monitoring, product delivery, surveillance, and reconnaissance. While there has been rapid progress in micro aerial vehicle design, locomotion challenges at the nano- and pico-aerial level, as in Figure 1, and low altitudes remain relevant and hinder the proliferation of this technology. Aerial locomotion is particularly difficult close to the Earth’s surface, where the winds can very rapidly change in speed and direction, rendering the conditions unfavorable for flight and for a planetary atmosphere such as Mars, where the density is very low compared to Earth. High levels of turbulence in the wind and even very low density such as that of Mars can be adverse for flight and poses severe flight-control challenges. In seeking solutions to these challenges, researchers have sought inspiration in biological flying systems such as insects and birds since they possess excellent flight prowess and can outperform current robots in nearly every facet of autonomous locomotion. Despite possessing miniature brains, natural flyers can solve complex tasks associated with navigation and flight control in an inherently complex environment. Flapping flight offers advantages over other platforms, especially at small scales, and it is the preferred mode of flight for natural flyers. Flapping flight provides high maneuverability while being collision-tolerant—traits that are critical for successful flight in highly cluttered terrain. However, due to the vastly different and highly dynamic nature of aerodynamic force production, wing actuation and flight control are incredibly challenging.

As per the latest research [2,3,4,5], the mosquito’s long, slender wings flap at a moderately high frequency relative to similar insects. Experimental and numerical studies reveal aerodynamic processes that have not been seen before in this type of flight environment. Additionally, with interest in developing insect-inspired robots for planetary studies, it is worth looking at the mosquito’s unique aerodynamic features for such an environment. These things make it necessary to look at this insect’s detailed aerodynamics and simulate its flight for Earth and a less dense Martian atmosphere. The application of biomimetics and bioinspired solutions to miniaturized-aerial-vehicle production has evolved to an incredibly small pico- and nano-aerial vehicle (PAV–NAV) size that exceeded its immediate predecessor, leaving a wide range of technology choices that MAVs need to sell. This review’s primary goal is to impart essential knowledge of the kinematics and aerodynamics of the insect-type flapping wing and membrane wing composition of mosquito and their suitability for planetary research.

An adult mosquito has three segments: head, thorax, and abdomen. The pair of wings and balancing organs, called halteres, are essential flight elements. Halteres, small ball-like structures, help mosquitoes to maintain balance during flight. For the imitation of insect-based aerial robots, the accurate identification of insect species is essential for wing design and venation. Identification of the organism is completed based on the wing pattern, and in mosquitoes, the species can be identified based on the size, shape, and color of the scales on the wings. The major dorsal part of the mosquito is called the scutum. In many mosquito species, the scutum might have very distinct scaled patterns used for identification [6]. Techniques such as wing geometric morphometrics (GM), artificial neural network (ANN) can be used for the identification and categorization of mosquito species based on wing shape characters [7,8,9]. The mass distribution in real wings is associated with venation patterns. Artificial wings having a pattern of veins are likely to be the ones that are biologically influenced and can be optimized to produce complex deviations close to those observed in natural ones [10]. Although wings are responsible for lift, haltere also plays an essential role in flight stability. If both halteres are immobilized, insects cannot remain aloft in flight. On all three axes, haltere has gyroscopic oscillations. By making rotations around three orthogonal axes, they produce distinctive angular velocity-dependent Coriolis forces. A single halter could therefore detect all rotations in space. In dipteran, halteres function as a micro-scale vibratory gyroscope [11,12,13].

**Figure 2:**
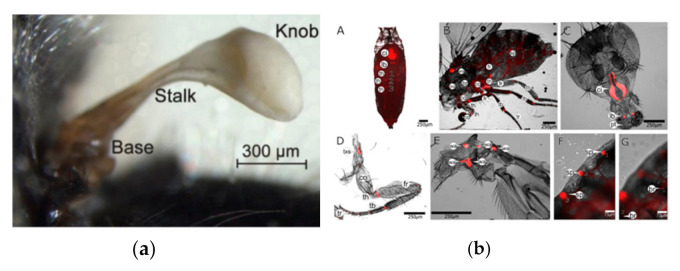
(**a**) Structure of haltere of a fly. Ref. [13]. (**b**) Resilin (red) localizes to distinct regions of the fly body. (**A**) pupa head, (**B**) legs, wings, and abdomen of an adult fly, (**C**) head, (**D**,**E**) several tiny Pro-Resilin-GFP patches legs, wings, and abdomen, (**F**) tracheal endings. (**G**) hair bases. Ref. [14], open-access article distributed under the terms of the Creative Commons CC BY license.

Experimentally, it is proven that the elasticity in the sensing direction of both the wings and halter structure is due to the presence of resilin, which restores the bending deformation. This rubber-like material covers many mobile joints and vein boundaries linked to the wing membranes of the wings. From a design point of view, recognizing the effect on the use of resilin and the entire resilome in biomaterial sciences, such as micro-robotics with elasticity advantages, would be very beneficial. The basic halter structure of the soldier fly is shown in Figure 2a, and the localization of resilin proteins in various parts of the fruit fly is shown in Figure 2b [13,14].

## 2. Wing Beat Frequency and Stroke Amplitude

### 2.1. Flapping and Actuation

Mosquitoes beat their long slender wings at an enormous speed with a flapping frequency in the range of 600 to 800 Hz, compared to their natural counterparts [2,3]. Computational and experimental studies have helped us understand the sources of high-force development in insect flapping wings so far, such as the flapping and deforming of fish fins and the integration of that knowledge into bio-inspired vehicle designs along with trade-off studies carried out during the bio-inspired design between efficiency and productivity [15,16]. The energy of a flapping wing MAV is vital in order to build flapping-wing aerial vehicles. It involves the design of an insect thorax-like energy storage mechanism in the flying vehicle, aerodynamic wing models based on blade element theory, optimizing the energy storage mechanism parameters using dynamic models to minimize the peak input power from the outer actuators throughout the flapping period [17]. For example, when designing a compact flimsy mechanism used for wing flapping, stability, controllability, and power dispensing are the main issues when size is reduced. The wing efficiency and advance ratio, controlled either by extending the stroke amplitude or increasing the flapping frequency, are the two most essential factors used in mimicking an insect. [18,19]. Wing flexibility also plays a role here. Compared to the rigid wing, it must be recognized that wing flexibility increases the capacity for thrust generation and performance for all kinematic patterns [20]. However, there is the development of insect-inspired robots with a frequency of up to 250 Hz on the micro-nano scale with different actuation systems such as piezoelectric, electromagnetic, and other actuators (see Table 1 above). The development of an actuation system that can generate a flapping frequency (600 to 800 Hz) close to mosquitoes is still a challenge. In this case, unstable aerodynamic processes need to be quantitatively determined over a broad Reynolds number scale to validate the morphological model test method [21]. For example, the mass and stiffness disparities along the wing of the blowfly give directions for designing a biomimetic structure in the case of insect-scale flapping wings [22]. Artificial wings must have biomimetic wing features similar to their natural counterparts to have substantial lift force. The artificial wing’s mechanical characteristics rely heavily on venation thickness, retaining a fully stringent arrangement during flapping motion and helping to generate appropriate thrust [23,24].

### 2.2. Stroke Amplitude

**Figure 3:**
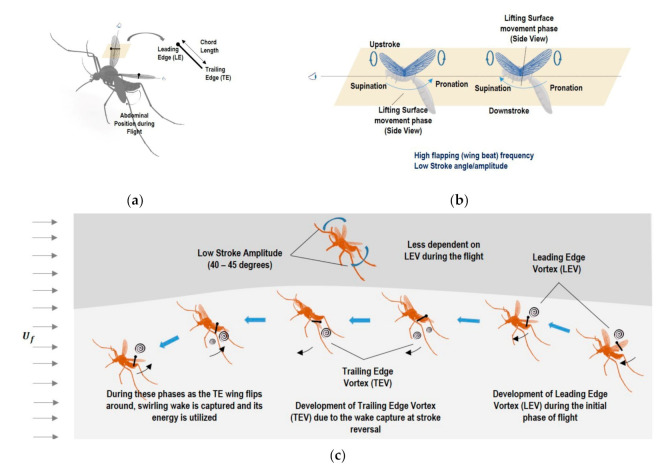
(**a**) Aerodynamic representation of mosquito during flight (**b**) Representation of mosquito-related flapping cycle phases (**c**) Graphical representation of formation of leading and trailing edge vortices during phases of the wingbeat cycle of mosquito flight.

**Figure 4:**
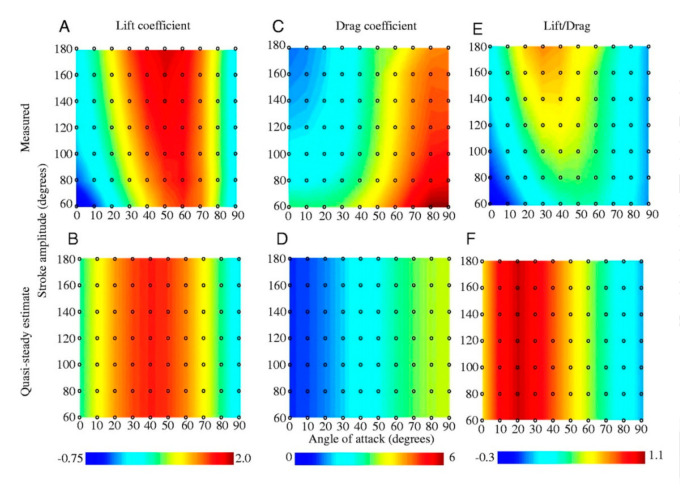
Stroke-based $C_{L}$, $C_{D}$, and L/D ratio in relationship with AoA and stroke amplitude. (**A**,**C**) and (**E**) have a mechanical model contour map based on the measured value. (**B**,**D**) and (**F**) showed measured values from the translational quasi-steady model using empirically measured force coefficients. Reproduced with permission from Ref. [25]. Copyright 2001, Company of Biologists Ltd. (Cambridge, UK).

Mosquitoes have other unique flight characteristics in addition to beating their wings at a very high pace. For instance, their long, slender wings move between the most anterior and posterior wing positions through a stroke amplitude of about 40 to 45° [3]. They have diminished reliance on LEV, which is opposite to the lift force generation mechanisms of other insects and animals such as birds and bats during wing translation [2]. Generally, for all hovering insects, including mosquitoes, there are four wing stroke phases: translational states called upstroke and downstroke, and rotational states called pronation and supination. Figure 3a–c show these phases as well as the complex aerodynamic mechanisms associated with mosquitoes. The accelerating wing experiences the accelerating fluid nearby as “added mass”. Table 2 shows the wing stroke amplitude and Reynolds number of hovering insects, including mosquitoes. Figure 4 gives the relationship of the lift and drag coefficients and lift-to-drag ratio with the angle of attack (AoA) and stroke amplitude. A and B give the quasi-steady mean lift coefficient ($C_{L}$) values, C and D give the mean drag coefficient ($C_{D}$) values, whereas E and F represent the lift-to-drag (L/D) ratio [25]. The mean lift rises with enhanced flap frequency, which proportionally increases the average wing speed and therefore lift, but as the amplitude of the stroke elevates, the mean wing speed and the lift also increase.

Nevertheless, the mean lift to the mean win tip speed squared ratio falls as the stroke rate rises, which affects the mean lift coefficient. It might be possible that the mean lift will escalate with a stroke amplitude square, vortex shedding impedes the potential outcomes of the mean wing speed increase [26]. Therefore, higher flapping frequencies are advantageous because there is an increase in the lift without a potential improvement in the lift-to-torque ratio. Although the mean lift and mean drag are influenced by varying the stroke amplitude, their relationship is linear [27]. Since the whole flow mechanics tend to alternate themselves as the stroke amplitude is varied, it anticipates that the mean lift-to-drag (L/D) and mean lift-to-torque (L/Q) characteristics would have a corresponding effect [27]. In mosquitoes, the low amplitude means 75 percent radial position of the lifting surface moves two chord lengths between the stroke reversals, which fails the fluid mechanic’s assumption about lifting surfaces acting as sweeping helicopter blades [2].

Mechanical hysteresis is another functional constraint; with the increase in flapping frequency, hysteresis can lead to a substantial loss of power [27]. Together with microelectromechanical systems (MEMS), the use of computational fluid dynamics (CFD) is a positive step towards understanding insect-scale flying robots [33].

Table 1 gives information about different bio-robotic models successfully developed in the frequency range of 30 Hz to 200 Hz. Vertical force enhancement is an essential factor in insect-like tailless flapping-wing micro air vehicles (FW-MAVs), and one such key challenge is that a lack of feedback control leads to instability after take-off. Insect mimicked aerial bodies are more challenging than birds due to different control principles [34]. Mosquitoes use their system as a motor during the leg push-off to control pitching torque [35]. To correct bias torques produced due to irregularities in the complex flapping mechanics of small insects, researchers have introduced a trimming system to correct bias torques that often lead to rapid free flight rotation if not adequately trimmed [36]. There is an observation that the bio-inspired honeybee and bumblebee wing configurations show optimal performances with similar wingspan and wing surface [37]. Although high-frequency flapping incurs higher inertial power requirements, it is vital for acoustic communication in mosquitoes [2]. A short rotation period or low stroke angle is generally associated with increased performance, a unique flying characteristic of the mosquito. The wing design and mechanism strongly influence the chosen aerodynamic features and performance [38]; for instance, the dry film based on a negative epoxy photoresist (SUEX) compliant flapping mechanism of the pico-air vehicle (PAV) airframe demonstrates excellent agreement with experimental results [39]. Fog or dew significantly affects flexible mosquito wings. Water accumulates on mosquito wings, folds them, and makes them useless for flight [40]. Essential observations from mosquito-related studies, such as in reference [4], showed that in the case of downstroke and upstroke, when the stroke amplitude is low, LEV does not play a role there, and delayed-stall could not contribute to force generation. Furthermore, wake from a previous stroke could be harmful to force generation. Reference [5] suggested that force peaks and stroke-related descriptions in Bomphrey’s [2] study were not explained clearly. A thorough investigation is needed further to understand the full benefits of high-frequency flapping and low stroke amplitudes in mosquitoes. Progression in the necessary technologies associated with insect-scale robots is mandatory despite many complex challenges [41]

## 3. Lift Generation Mechanisms

### 3.1. TEV by Wake Capture, Added Mass Effect, and Rotational Drag

**Figure 5:**
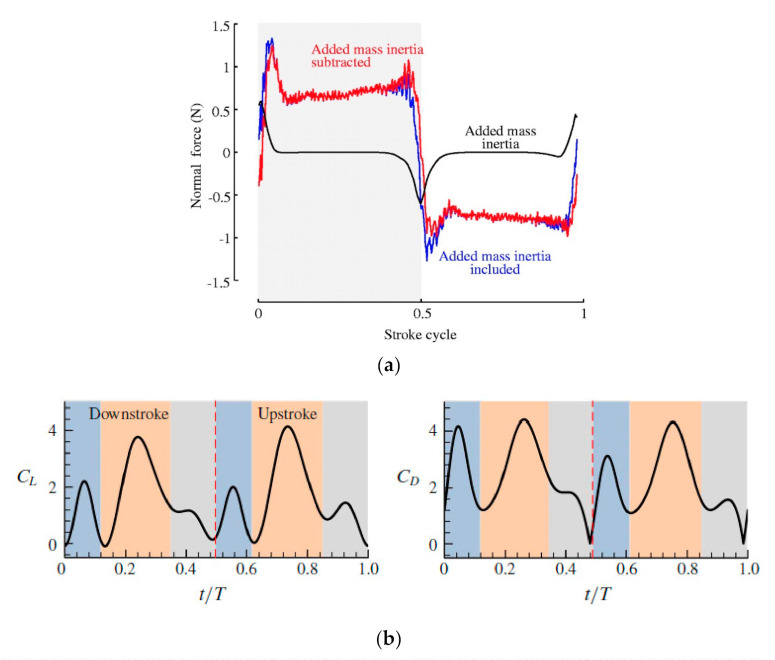
(**a**) How added mass inertia contributes to an estimated total aerodynamic force with a specific kinematic pattern at 45 degrees AoA. Reproduced with permission from Ref. [25]. Copyright 2001, Company of Biologists Ltd. (Cambridge, UK) (**b**) Coefficients of lift and drag in a single flapping cycle with a mosquito sample in flight. Reproduced with permission from Ref. [5]. Copyright 2020, Cambridge University Press (Cambridge, UK).

Wing-wake interaction or wake capture, a nonlinear, unsteady aerodynamic effect, significantly impacts the lift, required power, and dynamics of flight [42]. Mosquitoes possess several distinct aerodynamic characteristics among other counterparts: TEV due to wake capture; LEV, generally used for all insects of the same class; and most importantly, rotational drag [2]. TEV capture during the stroke cycle is a kind of wake capture because it depends upon induced flow during the half stroke. The parameter representing the unsteady effect is the Strouhal number, which is inversely proportional to stroke amplitude [5]. The Strouhal number (St) optimal range is: 0.2 < St < 0.4 for efficient flying [43]. The stroke amplitude of mosquitoes is low, which means an extremely high Strouhal number, so the flow throughout the wing is substantially unsteady. Reference [5] pointed out that in Bomphrey’s study in reference [2], the force peaks and their explanation were based on instantaneous streamline patterns that are very different from vortic patterns if the flow is substantially unsteady [5]. Furthermore, added mass, which plays a vital role in unsteady flow, was not explained well in Bomphrey’s study in reference [2]. As in Figure 5a, since the wing’s linear velocity is steady, added mass inertia seems negligible during much of the stroke. It contributes very little to the estimated aerodynamic forces [25]. In Figure 5b, t/T = 0–0.12, the inference is that there is a significant presence of force peaks, though there is no prior wake, which means that it is not wake-capture but an added-mass effect [5]. The rapid wing oscillation also contributes to significant added mass forces in the case of insects. Several studies related to fruit flies reveal that the added mass effect is a crucial aerodynamic mechanism. The Strouhal number, St, representing unsteady flows, explains such a mismatch. As the St surpasses 0.5, the added mass becomes dominant. For example, a Hawkmoth’s St is close to 0.315, lower than that of small insects, which does not dominate the added mass force but can interfere with the wing circulation [44]. Several studies demonstrate unsteady models that include quasi-steady, unsteady, circulatory, and non-circulatory flight, have limitations in the sense that they presume that their models comply with the condition of Kutta–Joukowski, which is not possible when the flow is all over the trailing edge as in the case of mosquito flight [45,46]. The advantage of rotational drag by mosquitoes during their flight is its intense contribution to lift. This lift contribution by the rotational drag should not be confused by the rotational lift, which is different.

**Figure 6:**
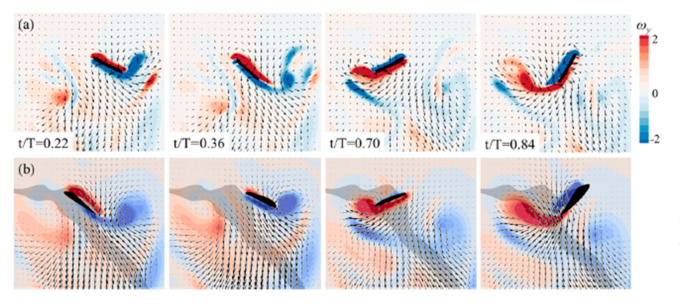
Instantaneous vorticity dissemination of the *y*-component at different t/T and the velocity in the symmetric *XZ* plane at the center point of the wingspan: (**a**) the numerical simulation results; (**b**) the PIV results from [2]. Reproduced with permission from Ref [4]. Copyright 2019, AIP Publishing (Melville, NY, USA).

Flapping frequencies have optimum ranges with two parameters depending upon higher AoA, low Reynolds number and leading-edge vortex development and shedding [47]. Wing rotation is significant in developing and reducing lift in flapping wing motion during the stroke period [48]. As opposed to traditional airfoils, insects usually flap their wings at higher AoA. A high Reynolds number can lead to spanwise flow within the vortex core which relies on wing shape and kinematics [49]. In some insects, such as dragonfly, while hovering, an unsteady force mechanism helps generate a new vortex ring, a downward momentum in the downstroke of either the hindwing or forewing, giving up upward force. The complicated relationship between the wing deformation and surrounding airflow has long prohibited the flexibility effect from being understood [50,51,52]. The time-varying vorticity flux distribution articulates the relation between the shedding vortex and force generation [53]. As seen in Figure 6, researchers used the immersed boundary method to model the 3-D flow field around a mosquito in hover based on direct computational analysis. The numerical computation findings were validated for the same results with the particle image velocimetry (PIV) measurements from reference [2]. At various intervals within a flapping phase, all the wing vorticity contours display good agreement [4], resulting in fully understanding the aerodynamic features such as TEV and wake capture. Sometimes, the forward force and a lift force component integrate to create the turning moment; meanwhile, the side force generates the restoring torque all over the manoeuvre alone [54]. Camber deformation is also essential. The dynamic stall can be delayed significantly, with the airfoil’s role having time-varying camber deformation, thereby delaying LEV production and shedding [55]. Camber deformation affects the aerodynamic forces on the flapping wing much more compared to a substantial twist [56]. Just after stroke reversal, the wake-capture mechanism accountable for a rise in thrust output decreased with rising downward velocity and fades away as soon as this velocity exceeds the mean wing-tip velocity [57]. Delayed stall, the rotational effect, and wake capture influence the surface aerodynamic properties of the flapping wings in forwarding flight. For instance, with an improved advanced ratio, the delayed stall effect deals with a rise in downstroke and fall in the upstroke. The rotational effect also relies on the advanced ratio and angle of the stroke. Wake capture is effective at an early upstroke rather than downstroke [58].

### 3.2. Wing Corrugation, Versatility and Other Factors

Flapping wing motion is often correlated with separated flow patterns, as flow separation on the wing surfaces has often existed [59]. The lift and thrust forces are also substantially responsive to flexural stiffness distributions, with optimum execution in various phase sectors [60]. In flexible airfoils/wings, the relative convection rates of positive and negative vorticity influence the thrust generation. The asymmetry between the LE and TE is also useful for producing thrust [61]. Wing corrugation is often assumed to play an important role as well. However, studies show that corrugation of the wing is intended for structural purposes, not aerodynamic ones. Corrugated wings have the advantages of being light and sturdy. These advantages are related to their aerodynamic properties and can be answered by further work in this field [62,63,64]. The contact between vortexes is the critical attribute that enables insects to produce sufficient lift to remain aloft. The standard unsteady vortex-lattice approach and the general kinematic model could be extremely reliable and systematic tools for aeroelastic studies in the future [65,66]. It is evident that the spanwise versatility of the wings improves the thrust marginally but reduces the performance [67].

It is important to note that wing-body interaction in flapping insects also substantially enhances the total lift production [68]. Wing deformation patterns induce a 30 percent increase in lifting force throughout the upstroke than the rigid wing model. For the flapping-wing flight, wing elasticity thus plays a fundamental role [69]. Compared to traditional rotary and insect-like flapping wings, flapping wing rotor (FWR) can perform reasonably well by generating a notably higher aerodynamic lift coefficient with power efficiency [70,71]. Mosquitoes produce the aerodynamic force to support their weight differently from their general counterparts, even though they use familiar separate flow patterns [2]. Wing aspect ratio, high flapping frequency, and small stroke amplitude of mosquitoes also allow high-intensity wing tones to be produced efficiently for acoustic communication [72]. Therefore, it is evident that due to vortices at the tip and root that interfere with the wing during the flapping period, unsteady lift and drag are produced. For assessing the performance of geometry and kinematic parameters of flapping-wing vehicles, power loading is more appropriate than the lift-to-drag ratio [73].

## 4. Unique Kinematic Patterns and Wing Flexibility

TEV through the wake capture method is a significant characteristic observed in mosquitoes at low Reynolds numbers as described in the previous section. A recent study reveals the total added mass as a factor here [5]. Mosquitoes advanced the traditional boundary of kinematic patterns. A substantial angular rate and exceptional stroke reversal timing are the two critical parameters that help the mosquito to generate the necessary force to support its weight during flight [2].

### 4.1. Kinematic Patterns

Flapping kinematics, aerodynamic modeling, and body dynamics are three primary building blocks of the dynamic flight framework [74]. To improve aerodynamic energy development, insects use time-varying pattern mechanisms during the flapping process [75]. Mosquitoes have a very precise axis of rotation despite lift produced due to rotational drag being dependent on the angular pitching rate by square. The wing’s pitching rotational axis moves from LE to TE amid pronation after the upstroke [2]. It is often better to combine aerodynamic studies with behavioral ones to understand flight locomotion in insects [76]. The study indicates that at different Re, vortex movement during its movement from LE to the wake, which allows for sustained vortex attachment, takes various forms [77]. Therefore, significant improvements have been made in predicting aerodynamic force mechanisms and power requirements in insect flight [78]. Methods of aerodynamic modeling are the most enticing for iterating rapidly across various design configurations. The span-wise movement of the LEV is a very significant function that most models do not notice. In order to understand the wing flapping mechanism’s efficiency, the modified quasi-steady 2D modeling is a good approach [46,79]. Insects can increase their flight strength by interacting with the contralateral wing during the dorsal stroke’s reversal (‘clap-and-fling’), affects the power loading, propeller efficiency and the metabolic activity in the aerial body [80]. At low Re, the spanwise flow appears almost more pronounced. An increase in the Reynolds number does not have severe effects on the LEV, so scaling up insect flapping is possible if the aspect ratio is below 10. Additionally, elastic deformation-based circulatory lift increase is the combined effect of an in-phase rise in wing velocity and wing camber shifts. [81,82,83]. The wing’s elastic deformation kinematics reveals that the incidence angle and the camber both display a reversal effect as they suddenly shift at the reversal of stroke. Even a primitive wing vein architecture is enough to reinstate the flexible wings’ capacity to produce forces at very close-rigid values. It is important to note that flapping produces stable LE vorticity at high angles of attack, continues over the stroke period, and raises mean aerodynamic forces. By modulating the TE’s flexibility and thus controlling the enormity of the vorticity of the LE, the magnitude of the generation of force can be regulated. [84,85,86]. Takahashi et al. measured the differential pressure distribution of different insect ornithopters and free-flying insect wings during flight phases such as take-off. Using micro differential pressure sensor developed using microelectromechanical systems (MEMS) technology they found that this measured distribution is characteristic aerodynamic force during the flight phase and proposed that this method combined other experimental techniques such as digital particle image velocimetry helps understand the unsteady aerodynamic forces. [87,88,89,90,91]. Experiments suggest that for substantial performance, the combination of a flapping phase with a feathering phase is significant in hovering and forward flight [92]. From the aerodynamic perspective, passive feathering gives lift development the necessary capacity at a very reasonable energy cost [93]. Digital particle image velocimetry, therefore, helps illustrate how flexible wings achieve aerodynamic strength. The phase delays in stroke movement of flexible wings impact the generation of a vortex, especially the leading-edge vortex (LEV), thereby supporting weight. The wake capture force is entirely unsteady during stroke reversal. For example, for dragonfly, the forewing LEV provides support for weight throughout the routine flapping flight [94,95,96,97,98] show a high dexterity level in wing motion [99]. This summarizes the LEV as the most significant aerodynamic element for most insects.

**Figure 7:**
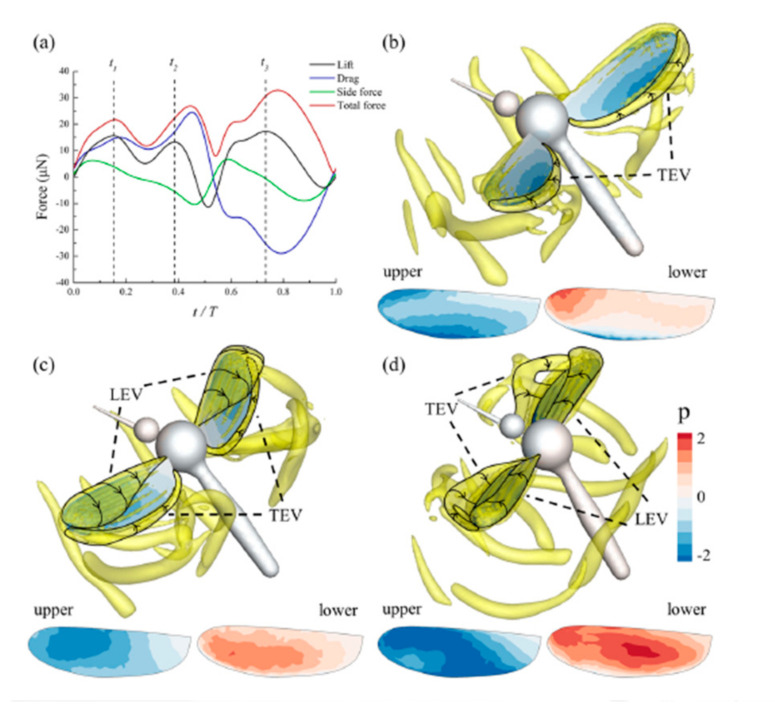
(**a**) Aerodynamic force assessment of a mosquito wing with a single flapping period. (**b**–**d**) The pressure contours showing distributions on the wing surfaces at particular *t/T* with instants *t*_1_, *t*_2_, and *t*_3_. Reproduced with permission from Ref. [4]. Copyright 2019, AIP Publishing (Melville, NY, USA).

LEV development is a function of the span, which means that separated flow in the wings’ outer regions or boundary exists, thereby clarifying free vortex simulation of wake progression [100]. Actual LEV configurations may be more complex [101]. At low Re, flapping type lifting devices have very high performance aerodynamically. Typically, the decrease in AoA during the upstroke with fixed AoA at the downstroke decreases the wake upstroke, notably reducing the effect of downstroke LEV production through wake capture [102]. To understand mosquito’s reliance on TEV and the role of delayed-stall, recent research on the mosquito kinematic model and a unique computational study with the immersed boundary method for mosquitoes is shown in Figure 7 [4]. In this study, for only one wing, the aerodynamic time history of the forces is plotted in Figure 7a, obtained directly from Lagrangian force integrated about the IB taken from [4,103]

(1) $$F_{Aero}=-\overset{}{\underset{\Omega }{\int }}F\left(s,t\right)\;ds$$

where Ω is the body surface represented by Lagrangian points, reference [4] observed some differences in lift force while having similar drag and side forces as compared to reference [2]. There are three lift peaks in the lift, shown by *t*_1_, *t*_2_, and *t*_3_ in Figure 7a, compared to four in reference [2]. The TEV developed at *t*_1_ binds itself and produces a broad negative pressure area at the upper surface TE, which leads to the primary lift point. *t*_2_ and *t*_3_ show both LEV and TEV’s presence, but pressure contours tell different stories due to distinct patterns on both the wing sides, which needs further examination, as in Figure 7b–d [4]. After the reference [2] study on mosquitoes in 2017, references [4,5] did some tremendous work on the unsteady aerodynamics of mosquitoes. These studies have explained the flying pattern and essential factors related to mosquito flight with computational and experimental aerodynamics. The study indicated that the delayed-stall mechanism has no direct relationship with aerodynamic force development in mosquitoes [5]. Early studies [49,104] also pointed to the delayed-stall mechanism’s irrelevancy for a lifting surface, having a low amplitude stroke [5].

### 4.2. Role of Wing Flexibility

**Figure 8:**
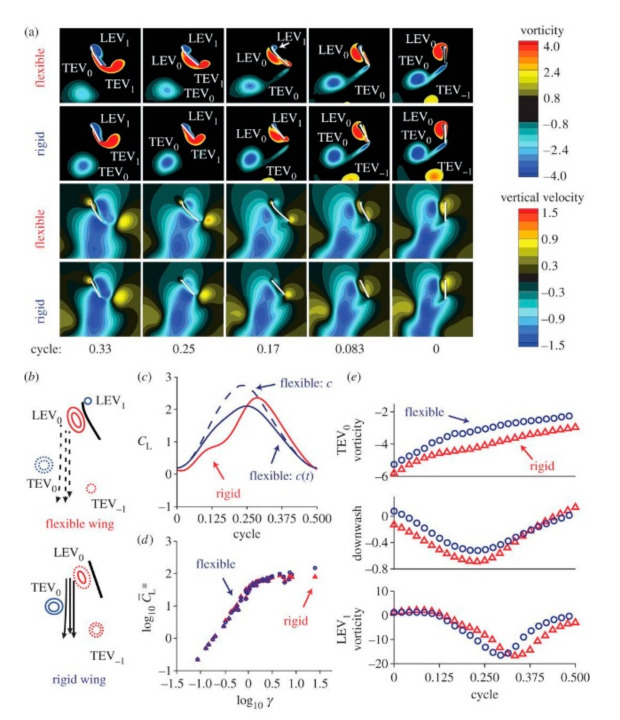
Results of streamlining for symmetrical rotation by analyzing the rigid rotating wing with *α*. (**a**) Vorticity and vertical velocity for rigid and flexible wings, (**b**) wing-wake interaction for rigid and flexible wings. (**c**) Time history of normalized lift (**d**) *C*_L_^∗^ relation with *γ* (**e**) Time histories of TEV, LEV vorticity with downwash. [105] Reproduced with permission from Ref. [106]. Copyright 2014 Royal Society Publishing (London, UK).

The flexibility of wings primarily leads to substantial lift generation, and the flight speed is significantly improved by gliding forces, indicating that the optimum layout of the wing structure and flapping motion may increase the efficiency of these vehicles. The size also affects insect hovering aerodynamics. Wing deformation is critical to the mosquito because it helps retain the LEV, i.e., delayed stall, therefore significantly reducing the overall aerodynamic strength needed for the insect to hover. The Reynolds number increases with increasing size, and so do the lift and power efficiency, which is why larger mosquitoes are more effective in searching and feeding [4]. A flexible wing can reshape its form, adjusting its camber to make the surrounding flow more effective. Figure 8 shows wing features at *Re* = 100. The translational lift with higher efficiency is used for flexible flapping, with low rotational force at stroke reversals. Figure 8c shows the lift’s variation from two peaks to one-peak shape at the stroke center. As the flexibility increases, there is lift enhancement characterized by *γ*, which is non-dimensional wing-tip displacement w.r.t. leading edge (Figure 8d) [105,106]. Flexible wings help in increasing the L/D ratio for superior performance. Though it generates lower lift and drag than the rigid, the chordwise deformation ceases the increase in effective geometric AoA, thereby changing the total resultant force direction upwards, increasing the L/D [107]. To better control and play with the dynamic characteristic modes, insects can utilize wing base flexibility [108]. Study shows that aerodynamic force affects the deformation of the insect flapping flexible wing, whereas inertial force controls deformation [109]. So it is clear that aerodynamic performance and wing flexibility have a peculiar relationship where the latter helps lift enhancement.

Flexibility also improves the propulsion efficiency by significantly reducing the rotation sequence losses [110,111,112]. In a flapping wing, wing flexibility is crucial as it demonstrates propulsive performance. [113]. Flexibility increases downwash in the wake, and therefore force. For kinematics, the reduction in tip LEV breakdown due to dynamic bending enhances force production before stroke reversal [114]. Flexibility, along with passive deformation, also significantly influences force production. High flapping frequency as observed in mosquitoes and high LE flexibility results in phase lag in the tip’s displacement and thus less vertical thrust production. Flexibility also affects trim conditions [115,116,117]. Structural mechanics and aeroelasticity are vital tools to understand insect wing flexibility [118]. The wing performs exceptionally well, considering wing flaps are resonant and density similar to insects’ natural wings for flexibility in hovering performance [119]. In its specific scope of chordwise flexibility, the flexible wing with proper LE venation can have more incredible aerodynamic performance [120]. So wing flexibility in bio-imitation and insect flight is an essential factor for modeling and imitating the bio-inspired robotic locomotion using soft organs, such as a general framework centered on a mobile multibody systems (MMS) model [121]. By utilizing a novel process called vortex trapping, elastic wings recycle energy from separated LEVs [122]. Using stereoscopic PIV on fruit fly, researchers found powerful axial flow components on the top wing surface and the axial flow in the vortex core of LE [123]. The general clap-and-fling effect fails to contribute to lift development and enhancement [124]. It has been found recently that elastic wing deformation also helps mitigate asymmetry in flapping in case of maneuvers [125]

### 4.3. Other Essential Factors for Kinematics

Compliant transmission mechanisms are a better replacement for rigid transmission systems to minimize total weight, reduce energy losses, and accumulate and liberate mechanical power during the flapping process [126]. The impact of the Reynolds number on LEVs around a wide range of size scales and modes, flexible structure performance with realistic models, turbulence studies in unsteady environments, and a systematic analysis on functional morphology to create real-life bio-inspired lifting surfaces and structures are some of the challenges associated with multimodal locomotion [127]. In imitating a mosquito-based robot, fabricating an actuation mechanism with a frequency range of 600 to 800 Hz with size limitations is a gruesome task. The insect kinematics that characterizes the natural insect flight is very complicated. The kinematic model enables this study using both the body and the stroke plane orientation of the insect in 3D space [128]. Spanwise wing deformation at stroke reversals often leads to mechanical energy loss in flight, even if aerodynamic power outshines inertial force [129]. To control aerodynamic forces and power, it is always better to take control over the angle of attack during the flapping process [130], for instance, a dual-differentiated four-bar flapping system for a lightweight vehicle with a tethered hover [131]. Regardless of the type, some insects, such as butterflies, have vortex rings developed over the wing while downward flapping, which grows from LE to TE [132].

From the previous section, we learned that dynamic wing pitching would significantly raise the thrust and thrust-to-power ratio while retaining the lift and lift-to-power ratio or increasing simultaneously [133]. Appropriate insect asymmetric strokes may boost the wing’s aerodynamic performance at low Reynolds numbers but may not function at moderate and high Reynolds numbers [134]. Wake deformation is often most extreme behind small lower aspect ratio wings, meaning that the insects that fall in this category are reflected as substantially risky in terms of measurement error when there is a shortfall of the distance between the wings [135]. Here, the non-uniform downwash effect leads to induced power factor, k, contributed by chord distribution and the advanced ratio [136,137]. Interestingly, if the insect wing beats at a high frequency, such as mosquitoes, and has a short wing length, the wing’s relative velocity is minimal. As a result, the moderate wing lift coefficient is relatively high to balance the weight, far higher than that of cruising aircraft [138].

**Figure 9:**
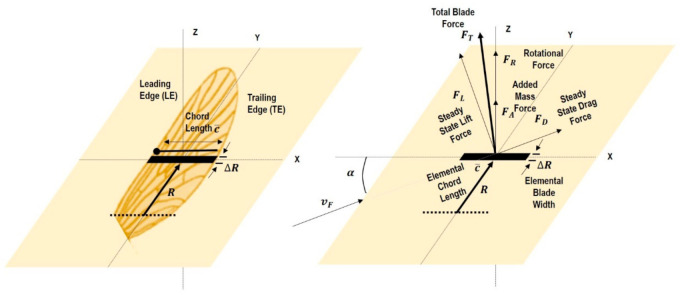
Quasi-steady wing (or blade) element theory modeling of mosquito wings. Reproduced with permission from ref. [144]. Copyright 2016 Company of Biologists Ltd. (Cambridge, UK).

In the case of insect-based robotics, UBET (unsteady blade element theory) provides reasonably good estimates of the thrust developed by the wing flapping systems by comparing estimated thrust with measured thrust [139]. Using unsteady blade element theory, researchers have shown that for the evolution and building of FM-MAV, perfect twist configuration can be obtained from wing root offset of 0.20$\bar{c}$ as far as flapping wings are concerned. The power loading is just two percent greater for the positively twisted wing than the full force-generating flat wing. For immense power loading, force/power ratios, it is desirable to opt for a high-frequency flapping wing using the geometric AoAs [140,141,142]. As per the quasi-steady wing (or blade) element theory-based aerodynamic model given in reference [143,144], various forces act on the wing, which can be taken into account during modeling. The total force acting on the blade (a spanwise division of insect wing into finite blade elements) can be estimated as the addition of rotational, steady-state, and added mass forces, as shown in Figure 9.

(2) $$F_{T}=F_{L}+F_{D}+F_{R}+F_{D}+F_{Wake\;Capture}$$

(3) $$F_{L}=\frac{1}{2}\rho \bar{c}\|v_{F}\|{}^{2}C_{L}\left(\alpha \right)\Delta R$$

(4) $$F_{D}=\frac{1}{2}\rho \bar{c}\|v_{F}\|{}^{2}C_{D}\left(\alpha \right)\Delta R$$

(5) $$F_{R}=C_{R}\rho \dot{\alpha }{\bar{c}}^{2}\|v_{F}\|\Delta R$$

(6) $$F_{A}=\frac{\rho \pi {\bar{c}}^{2}}{4}\left[\frac{v_{F}\cdot \dot{v_{F}}}{\left\|v_{F}\right\|}\sin\alpha +\|v_{F}\|\dot{\alpha }\cos\alpha \right]\Delta R$$

where $C_{R}$ is the rotational force coefficient having specific values for each insect wing and $v_{F}$ is the instantaneous flow speed in the elemental plane [143]. It is essential to consider how the aerodynamic model assumptions affect optimal kinematics of the wings during hovering. Rotational motion produces lift with low power consumption compared to translatory [145]. The contribution of the vertical force mostly produced during the downstroke becomes more dominant as the flight speed increases [146]. The flight efficiency of insects in free flight is very significant for the research of bionic fluid mechanics. [147]. In big animals such as the calliope hummingbird, during the wing-beat process, both the downstroke and upstroke produce significant thrust for drag reduction, but such thrust output comes at the price of induced adverse lift at the time of upstroke [148]. Rotational acceleration developed at the end stroke during the flapping (LEV) significantly reduces lift [149]. Optimization of the power output during floating flight can be achieved by knowing the mandatory optimum pitching axis for flapping wings, which saves around 33 percent of the power during hovering [150]. For design purposes, remember that soft vein joints in passive deformation improve the chordwise flexibility and work well [151]. As far as propulsive performance is concerned, propulsive characteristics are significantly affected by the phase angle and mean wing spacing in the flapping wing [152]. Most insects combine their fore and hind wings to produce substantial lift. However, synchronously flapping two wing pairs together tends to create extra lift force [153]. Compared to gliding flight, the anticipated power savings can be influenced by flapping wings in the ground effect, depending on the wing motion [154]. As the size of the insect decreases, the impact of air viscosity on insect wing movement increases [155]. The scale-dependent distribution of energy in the turbulent ambient flow is an important element in how aerial insects such as bumblebees regulate their body orientation. Similar to mosquitoes, bumblebees use unsteady aerodynamic mechanisms, for example, LEV generation, wake capture, and rapid end-of-stroke rotation to enable them to fly [156,157]. Bumblebee-based miniaturized drones are all ready to fly to Mars very soon.

For tiny aerial insects with a low Reynolds number (Re), such as mosquitoes, viscous effects are more. These insects typically switch their flapping mode to overcome this problem; for instance, the planar-type upstroke to deeper U-shaped upstroke is used to generate large vertical forces [158]. During hovering, the wing with phase flap (alula) provides the maximum lift but the lowest performance and a stabilizing effect on the LEV [159]. For a flapping drosophila, at high stroke amplitude, a hairpin-like vortex loop and stroke reversal affect the instant time the wake capture materializes [160]. Insects even compensate for wing damage. Researchers discovered that insects such as the phorid fly compensate for the loss by increasing the amplitude of the stroke and the angle of deviation [161]. The lift is generated during upstroke for hovering flight due to stable LEVs and a stronger downwash at downstroke [162]. Finally, this entire section summarizes that TEV is a dominant part of mosquito flight, while similar insects, e.g., fruit flies, have comparable sizes, and Reynolds numbers depend on the LEV. This distinction is due to the mosquito’s significantly low stroke amplitude as a unique kinematic characteristic. As described above, passive deformation affects the maximum aerodynamic power but takes care of the delayed stall. With the increase in Reynolds number, the efficiency and performance of mosquitoes also increase [4]. At Re below 70 for miniaturized insects, there is a rapid effect on lift and drag due to the viscous effect being very high [163]. The Reynolds number, which has an inverse relation with $k=\pi fc/U_{ref}$, reduces the frequency. The aspect ratio and the flapping amplitude are factors affecting k, and the physical size variance of mosquitoes is a significant factor in the variations of their Reynolds number. As a result, with a Reynolds number increase, the lift coefficient and therefore flight efficiency is enhanced, i.e., larger, which may be the explanation for why larger mosquitoes are particularly good feeders [4].

## 5. Improvement in Future Wing Architecture and Miniaturized Drone Designs

Recent advances in small-scale manufacturing and control have made it possible to build insect-scale robots. Nevertheless, there are still numerous constraints on component technologies, such as scalable high-energy storage, which restrict their functionality and propulsion, power, and control architecture [164,165]. Insect robots have not yet demonstrated characteristics such as the ability to traverse complex and substantially dynamic habitats, rapidly adjust flight speeds and even directions, the robustness to environmental threats, and the ability to travel long distances autonomously instead of their natural counterparts. Can mosquitos’ specific aerodynamic features help improve wing architecture and miniaturized drone designs in the future? The answer to this question needs more intensive research, but as per the existing literature, we observe:

### 5.1. The Importance of Dynamic Stability, Mechanisms, and Mathematical Modeling

**Figure 10:**
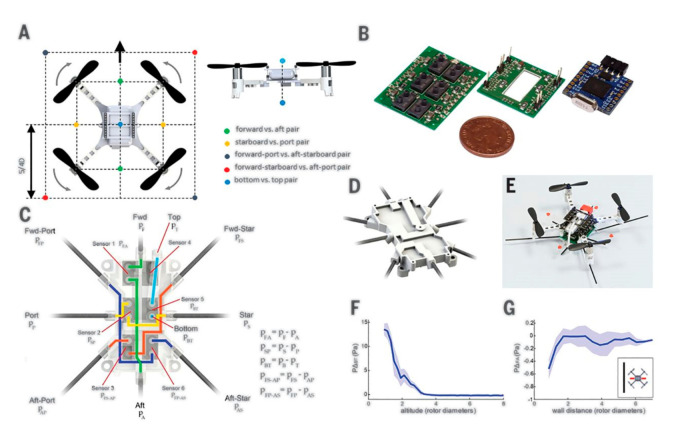
A quadcopter-based model system to experimentally verify the mechanosensory collision-avoidance mechanism in mosquitoes. The copter has (**A**) pressure probes for maximizing deltas near proximity, (**B**) pressure sensing components, (**C**) schematic diagram of the copter (**D**), tube network of sensors, (**E**) real flying prototype, (**F**,**G**) differential pressure with close proximity to the ground. Reproduced with permission from Ref. [166]. Copyright 2020, AAAS (Washington, DC, USA).

Free of a radial position, the aerodynamic force is extremely positive regarding lift interceded by rotational drag. It covers the entire wingspan, incredibly close to the root, where the lift is negligible. This specialty, including lower inertial costs and smaller pitching torques, is possibly crucial in creating the mosquito’s high aspect ratio wings [2] and is one of the design factor in imitation. Apart from having excellent aerodynamic characteristics, mosquitoes also possess good dynamic stability, a boon for the future design of miniaturized drones. Mosquitoes have a unique behavioral characteristic related to the response to avoid any obstacle during the short-range, very blurred vision, and change direction in flight called mechanosensory collision-avoidance mechanism. Researchers use CFD-based dynamic kinematic analysis to measure corresponding changes that appear at the pressure and velocity cues at this mechanosensory antennae. Figure 10 shows the model quadcopter fitted with such sensory equipment. By detecting nearby obstacles during flight, the model system successfully emulated the mosquito model’s behavior [166]. Insects typically take-off from the ground using a catapult technique to impel legs against the ground surface while propelling them into the air using their pairs of flapping wings [167]. Although mosquitoes use a slightly different technique, such as flapping their wings before jumping, this combination is an effective way to get rid-off unspecified terrain or steer clear of enormous obstacles, and even the host will not detect them.

Not just wings but fog and dense gas affect stability by increasing the aerodynamic drag on halteres [168], so of course, the knowledge of mechanisms is a must at these scales. As it is evident that smaller stroke amplitudes such as that of mosquitoes have strong unsteady effects, high flapping frequency in insects can be explained appropriately using the rigid body assumption and vice versa [78,169]. To understand the dynamics of these miniaturized insects and use it for mimicked robots, mathematical modeling or CFD is a great tool. The aerodynamic force and moment generation in insects is oscillatory due to unsteady flow, so mathematical methods that deal with nonlinearity should be used [170]. Kinematics can be easily imitated for real insects, but artificial insect-based robots are not easy to build. Mathematical models must also be simplified [171].

### 5.2. Transmission Systems, Suitable Controllers, and Acoustics

Sensory and biomechanical systems must be taken care of in order to emulate responsive insect bio-robots. Current gliding animals have used these to track and guide their descent and are an essential factor in the evolution of modern biomimicked flight [172]. One such key challenge for designing and producing insect flapping-wing robots is generating a productive and efficient transmission system to control flapping wing movements. Insect thoracic based system actuated by electrostatic force [173] or bio-inspired thoracic robotic designs for generating kinematics of asymmetric wing almost similar insects found in nature are some examples [174]. Table 1 gives light detail about some of these mechanisms. Even suitable controllers are needed to control the motion and look into stability and performance. The development of real-time controllers that can use the concept of an input–output linear time-invariant (LTI) equivalent system to apply the desired trajectories to micro-robotic insects is an example [175]. As artificial muscles can withstand the stresses caused by collision impacts, they are a good alternative to actuation. However, due to nonlinearity and limited bandwidth, these soft actuators have yet to show adequate power density for lift and are not suitable for flight control [176]. Researchers have designed stable robots using soft artificial muscles made from multi-layered dielectric elastomer with a resonant frequency of 500 Hz, recently in 2020 [176]. In a low Reynolds number flapping wing, the dynamic stall is a vibrant, dynamic problem. It accompanies many characteristics such as dynamic stall vortex, large aerodynamic loads that can mix with structural dynamics, and even negative damping [177]. The transmission system’s total weight was substantially decreased by piezoelectric transmission [178], which is still being experimented with so far in several versions. As discussed in the previous section, elastic wing properties are also very significant. Material analysis such as flexibility and compatibility in designing realistic wings, along with the transmission mechanism, and good elastic properties are fundamental [179]. Research also shows that bio-inspired insect robots undergo a stabilization technique called vibrational stabilization, with exceptionally high frequencies [180]. Without the need to use a thrust force, some insect species generate the flapping lift sufficient to retain their body weight [181]. Researchers found that the CG repercussion on longitudinal flight stability is a common characteristic of all tailless flapping insect species, with research restricted to the longitudinal direction [182]. It is necessary to know that the active control mechanism, partnered with light microcontroller-based actuators, can produce significant control torques to keep the robot airborne [183]. High sweep amplitude is more beneficial for power requirements than low amplitudes that need higher frequencies, resulting in higher inertial forces to generate a similar vertical force [184]. The modern Robobee from Harvard University or Robofly by UoW had been manufactured with proven MEMS piezo-based mechanisms, powerful miniaturized controllers, and laser power and at a large scale, reconfigurable multi-rotor using a novel active-passive motor scheme have already been proposed [185].

Have you ever wondered if there is an aerodynamic buzz or pitched whine associated with mosquito flight? This is due to the high flapping frequency. When it comes to insects such as mosquitoes, the acoustics are crucial. Researchers have tried to figure out why insects, particularly mosquitos, have this peculiar trait. In order to better understand the sound generation mechanism of flapping wings, Sueur et al. (2005) discovered that the flapping sound is directional, with the wing beat frequency dominating the front and the second harmonic dominating the two sides [186]. Although it is clear from the preceding sections that flexibility plays an important role in lift generation, its impact on aerodynamic sound is not well understood. The fluid–structure–acoustics interaction of flexible flapping wings was numerically investigated using an immersed boundary method at a Mach number of 0.1 in a recent study published in 2019 and 2020. There were three important observations made; (1) Coupled (translating and rotating) motion produces smaller sounds than the translating wing, and greater rotational angles convert the dipole sound to a monopole sound. Sound fields are present, but they shift downstream for large flexible wings. (2) The flapping frequency dominates the sound; (3) when the wing is flapping with a stroke plane angle less than 90°, the sound on the windward side is noticeably louder [187,188]. This explains, to some extent, the extreme buzz produced by mosquitoes with stroke amplitudes in the range of 40 to 45° and high flapping frequency (~800 Hz).

For the information of our readers, immerse boundary method (IBM) is a numerical technique by Peskin in 1972 [189] that deals with the boundary conditions for grids that do not conform to immerse boundary shapes [190]. Mittal et al. developed sharp immersed IBM to analyze incompressible viscous flow past 3D immersed bodies, for example. It manages complex immersed surfaces described by Cartesian grids using the ghost-cell methodology to satisfy boundary conditions. [191]. However, researchers are developing the immersed boundary surface (IBS) process, based on active cell concept. In terms of numerical investigation of the aerodynamics of insect-like complex geometries, IBM techniques are effective.

## 6. Flight in a Low-Density Environment Such as Mars

A low-density atmosphere such as that of the red planet Mars has an average pressure of 0.6% of Earth’s. Mars also has unearthly features, such as carbon dioxide (CO_2_), the primary atmospheric variable, which condenses in the Martian polar regions and the middle atmosphere [192].

### 6.1. Prior Work

According to the Russian Academy of Sciences, during an experiment on the ISS, natural mosquitoes could live in outer space, incredibly for 18 months, and could be taken back to Earth alive. Studies indicate that the fixed-wing drones capable of flying in the future fly on Mars shall have poor performance than other solar entities because of low Reynolds number values. Drones flying in a lower density environment, such as Mars, relative to Earth, stop providing the needed positive performance [193]. As far as planets such as Mars are concerned, insect-like compliant wings for low-density environments improve aerodynamics and low power design. High lift coefficients can be obtained by looking into strict dynamic similarities between the bio-inspired insect flight regime and the Martian climate. Due to the extremely low density on the red planet, there is an influence of inertial power. Minimal flight time is the greatest challenge for flapping-wing micro-air vehicles because of restricted onboard energy storage space. Given the average ground solar spectral irradiance and efficiency of solar cells in terms of energy conversion, the difference in the energy supply rate by size is assessed [194]. The flapping-wing design for the Martian atmosphere showing the modeling and simulation of a micromechanical flying insect (MFI) flapping-wing called Entomopter is built for the Martian atmosphere for continuous and autonomous flight. The overall geometry is built on hummingbirds and large insects for this micromechanical insect. Due to the very low density, unsteady aerodynamics of flapping wings need an investigation [195]. DelFly and ExoFly are the two examples of flapping winged flying robots designed particularly for low-density flight. Although various physical characteristics require adaptation to Mars conditions, studies reveal that this should not be a significant obstacle to feasibility [196]. Marsbee is a bumblebee imitated robot with enlarged wings for the vehicle to sustain weight in the Martian atmosphere. Just like the bumblebee, the wing-to-body mosquito mass ratio is just 0.52 percent. A consistently large change in the wing area raises the overall weight by just a fraction. The excellent ability to hover, fly efficiently even under the impact of forces, and work at fast forward speeds with an exceptional aerodynamic capability makes it an appealing biomimicking candidate in both cases [197]. In 2020, NASA sent the *Perseverance* rover on a Mars mission with the *Ingenuity* helicopter, which landed safely on February 18, 2021. This rotorcraft has a rotor diameter of 1.21 m and its performance at a low Reynolds number has to be assessed. The insect-based flapping wing can easily overcome these difficulties in this rarefied atmosphere. Insects have an excellent capability to use unsteady aerodynamics mechanisms in such low values of Re [198].

### 6.2. Flight Feasibility in a Low-Density Environment

For mosquito-based robots’ suitability for flight in low-density environments such as Mars, it is essential to account for the lift enhancing unsteady mechanisms such as wake capture, rotational drag, and added mass effect, perfect for the flight on Earth. This part of the review is purely based on aerodynamics, but it is essential to note that the actuator dynamics and materials used for such mimicked robots in such an environment also play a significant role and are a subject of research. To date, the study has only focused on wing beat motion to generate enough lift to sustain weight in such an environment. Once this question is appropriately answered, one can design the sensors, actuators, controllers, and power sources for success on Mars. The findings reveal that there are four major challenges to overcome in order to successfully fly through the Martian atmosphere. Due to the high concentration of CO_2_ in the atmosphere, traditional oxygen air breathing motors cannot be used; instead, we must rely on chemical or electric propulsion, which is difficult with insect-sized robots. Second, because of the low density, it is difficult to generate enough lift to fly. The third issue is Martian gravity, which is one-third that of Earth, and the fourth and most intense issue is temperature, particularly at night, where it plunges to around as low as −90 °C, making it difficult for components to survive if left unheated. As per references [197,198], who have extensively studied this part of the flight, the following can be possible, which authors of this review have compared to mosquito flight too.

**Figure 11:**
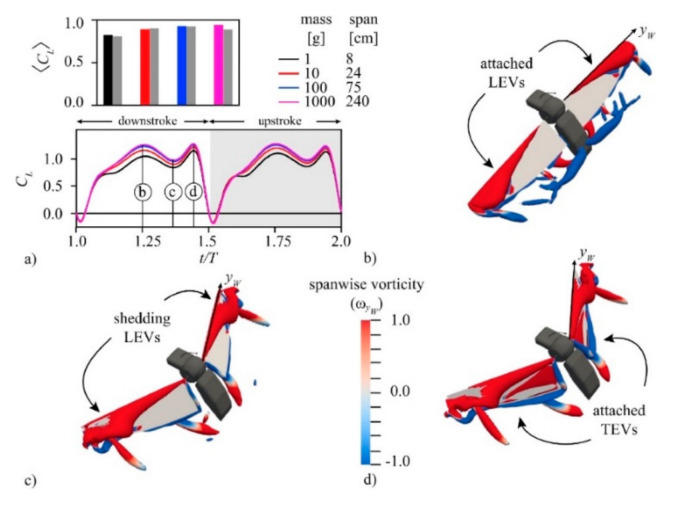
3D Navier-Stokes solutions asked on Q-criterion (**a**) $C_{L}$ coefficient and time histories (**b**) span-wise vorticity contours for LEVs, high $C_{L}$ (**c**) LEV shedding and reduction in lift (**d**) enhancement using the rotational lift (TEVs). Reproduced with permission from Ref. [198]. Copyright 2021, Elsevier B.V. (Amsterdam, The Netherlands).

**Figure 12:**
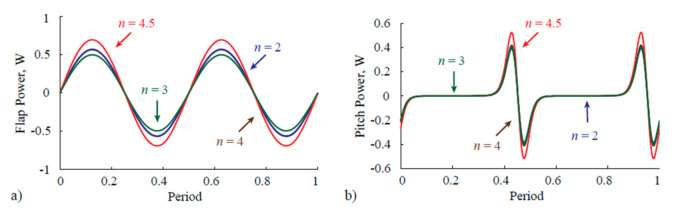
Time histories of the (**a**) flap power and (**b**) pitch power required for hovering on the red planet with different wing sizes *n*. Reproduced with permission from Ref. [197]. Copyright 2018, IOP Publishing (Bristol, UK).

1. Studies on bumblebee-type insect characteristics for Martian flight show that in order to achieve hover on the red planet, it is crucial to offset the reduced density and reduced gravity by making adjustments to the flapping motion. This offset can be achieved by wing scaling without changing the aspect ratio (though mosquitoes have a higher aspect ratio than bumblebees). Since the flapping amplitude is not changed, reduced frequency k will not be affected. However, this should impact the Re, which is again minimal because of density. Studies also suggest that a high flapping frequency is needed to offset the density and gravity factors. The wingtip Mach number also plays a significant role here. Mosquito imitated robots fall in the excellent category because mosquitoes have a very high flapping frequency with a low stroke amplitude, both of which are essential factors in determining Martian flight and need further investigation. The study on wing scaling on bumblebees comes with high power requirements, but it is possible that with mosquito-mimicked robots, there will be a relief in that matter because mosquito already flaps at a frequency beyond 800 Hz, which is close to the requirement (~990 Hz) as per reference [197] for offsetting the reduced density and gravity. So, wing scaling need not be as close as for bumblebee. Research is in progress in this regard.
2. Reference [197] also gave light about the aerodynamics related to MAV’s on Mars. Despite a low Reynolds number, the lift enhancing mechanisms such as a delayed stall, rotational lift, and added mass effect could help produce sufficient lift. From reference [198], the study published in 2021, the vorticity contours and lift time histories, as shown in Figure 11, established based on Q-criterion and coherence in vortex structures (low-pressure regions), show that the average lift is relatively high for weight balancing and time histories are similar to insects on Earth, as shown in Figure 11a. Due to LEV, the $C_{L}$ value is high because of the lift peak during each half stroke, as in Figure 11b. It can be linked to the negative pressure gradient due to high vorticity near LE. When the stroke ends, the reduction in $C_{L}$ due to the shedding of LEV is taken care of by rotational lift using attached TEV, as shown in Figure 11c,d. Mosquito flight works in almost the same way due to their unique aerodynamic mechanisms discussed in previous sections and TEV use for lift enhancement.
3. It is essential to note that wing scaling is associated with a penalty related to actuation power. In that case, the inertial power is more significant than the aerodynamic one because of ultra-low density. Figure 12 shows the flap and pitch power time histories required for hovering on the red planet with different wing sizes n. Because the flapping amplitude and frequency changes to obtain equilibrium as n increases, wing kinematics must be optimized to minimize the power requirement [197].

Interestingly, power contains significant negative stoke values, and amplitude rises with the wing size [199]. Missions such as Marsbee, with a broad testing range from 10 to 100 km and above, will enable low-altitude, substantially high-resolution imagery of Mars and in-depth long-range Martian exploration will allow the observation and study of Martian atmosphere, and phenomena such as dust storms [200].

Insect-inspired robots for planetary research are a booming new area of research. Work is in progress, and a few pieces of literature are available to help. Weight is also a factor affecting performance. There will be a difference in the masses/weight of drones on Mars than Earth due to gravity on the red planet. The weight is decreased by 61.5 percent [1]. Mosquito-based robots have many factors associated with being effective in low-density atmospheres such as Mars. For instance, as it has low stroke amplitude and high flapping frequency, and considerable wing size, which is associated with necessary power and stability, it can hover with stability.

So, it is clear that low Martian atmospheric density makes it difficult for flight on Mars. Aerodynamic forces mostly rely on the atmosphere’s density, which restricts conventional aerial configurations on Mars. In the simulated Martian environment, trimmed flight and hovering are only feasible if insects’ dynamic resemblance on Earth is achieved. This can be accomplished by maintaining the necessary dimensionless parameters by scaling the wings to three to four times the standard size, as described above. Due to its ultra-low density, the maximum power available is because of the inertia of the wings. By using a torsion spring, the inertial flap strength can be significantly reduced [197]. When developing potential bio-inspired robots for planetary studies, all these considerations need to be taken into account. However, mosquito-based characteristics modified according to the Martian atmosphere are an excellent alternative.

## 7. Conclusions and Outlook

Mosquitoes are without a doubt among the best flyers and intelligent insects on the planet. The study of this insect’s anatomy, kinematics, aerodynamics, and stability has so far revealed the value of imitating it, creating smart tiny drones, and using them for anti-disease, atmospheric studies in low-density environments such as Mars, and many other applications. Here are the conclusions from this review:

1. Since mosquitoes have extremely unsteady flow around the wings, the aerodynamic mechanisms are way different from the same group of insects. A high flapping frequency and low amplitude stroke are beneficial in terms of flight, attraction, and feeding. Mosquitoes extensively take advantage of rotational drag and TEV through wake capture for lift enhancement and even sustaining their weight during flight.
2. Wing deformation and the Reynolds number are two crucial factors influencing the flight. Wing deformation is essential to the mosquito because it takes care of delayed stall, reducing the overall aerodynamic energy required for hovering flight. Compared to insects with substantial stroke amplitude, mosquito lift-related output is controlled by various aerodynamic processes. However, most of these insects create a lift with the support of the LEV, which binds and advances along with the wing and the corresponding vortex movement. Flow around mosquito wings is exceptionally unsteady and generates lift by mechanisms such as TEV using wake capture, which is now replaced by the added-mass effect for mosquitoes in particular as part of the new study and the ‘rapid-pitching-up rotation’ mechanism.
3. As per the analysis, a sizeable aerodynamic force is produced when the rate of change of time at the first instant of vorticity is affected by the rapid production of opposite-sign vorticity at distinct wing locations. Big mosquitoes are active in feeding hosts because increasing size correlates to an increased lift coefficient and power efficiency, increasing the Reynolds number, and thus increasing aerodynamic performance.
4. The excellent ability of a mosquito to hover, fly efficiently even under the impact of forces, and work at fast forward speeds with an exceptional aerodynamic capability makes it an appealing biomimicking candidate for investigating low-density planetary atmospheres such as that on Mars. Mimicking a mosquito-based miniaturized flapping-wing insect robot for planetary studies comes with several challenges. For example, in an extremely low-density atmosphere, there is a drastic impact on the flight’s efficiency and sustainability. Wing length is also one of the factors that plays an essential role in-flight stability.
5. All the aerodynamic features examined in this review support the ability of mosquito-imitated robots to fly in low density that needs to be thoroughly explored experimentally and controlled in flight. Despite the challenges, mosquito-imitated robots present a bright and auspicious future.

This review paper hopefully provides valuable information for further investigation and in the elaborative study of unsteady aerodynamics related to mosquito flight, kinematics, and low-density environment, which will help to successfully imitate these insects for various applications. The future work will be the production of the mosquito-inspired robot with suitable materials and improved aerodynamics.

## Figures and Tables

**Table 1:** Some insect-based miniaturized systems with flapping frequency range 30 ≤f≤ 200 Hz. Reproduced with permission from Ref. [16], Copyright 2019, Elsevier B.V.

Flapping Wing System	Year	Developed by	Mass (g)	Wings	Span (mm)	Frequency (Hz)	Actuation System
Nao Hummingbird	2007–11	AeroVironment Inc.	19	2	165	30	DC Motor Driven
KU Beetle	2009–C	Konkuk University	21.4	2	160	30.5	DC Motor Driven
Pudue Hummingbird	2011–C	Purdue University	12	2	170	30–40	DC Motor Driven
3D Printed Mech Insect	2011	Cornell University	3.89	4-X	143	30	DC Motor Driven
Dipteran	2014	NTU Singapore	3.51	2	100	33	DC Motor Driven
KULibre	2015–16	KU Leuven	3.39	2	50	40	DC Motor Driven
MFI	2001–07	UC Berkeley	0.1	2	25	100–275	Piezoelectric
RoboBee	2007–C	Harvard University	0.1	2	30	110–120	Piezoelectric
FW-MAV	2011	Air force Tech	0.35	2	70	30	Piezoelectric
Insect Inspired F-MAV	2012	US Army Res Lab	0.03	2	2.5 *	156	Piezoelectric
LionFly	2013	Penn State, USA	0.112	2	45.78	37	Piezoelectric
Insect FW Robot	2017	S Jiao Tong University	0.084	2	35	100	Piezoelectric
Bioinspired FWMAV	2018	Toyota R&D Labs	0.598	2	114	120	Piezoelectric
RoboFly	2018	UoW, Washington	0.19	2	13 *	170	Piezoelectric
RoboBee	2019	Harvard University	0.09	4-X	35	200	Piezoelectric
OVMI	2008–C	Valenciennes Univ	0.022	2	35	80	Electromagnetic
FW-MAV	2011	KAIST, South Korea	2.86	2	75	68	Electromagnetic
FW-MAV	2013–16	Purdue University	4	2	86	90	Electromagnetic
FW-MAV	2017	Beihang University	0.093	2	30	101.4	Electromagnetic
Flapping Wing Platform	2018	Beihang University	0.05	2	56	35	Electrostatic

C-Current, * Wing Length, MFI: Micromechanical Flying Insect, f: Flapping Frequency.

**Table 2:** Dorsal and Lateral motion characteristic of four insects.

Name of the Insect	Average Weight (mg)	Re	Aspect Ratio	Flapping Frequency (Hz)	Reduced Frequency (k)	Wing Stroke Amplitude	References
Mosquito	2.1	120	4.2	600–800	~0.559 *	44°	[2,28]
Fruit fly	0.96	130	2.7	218	0.212	140°	[2,29,30]
Honeybee	86	1100	3.2	~240	0.244	91°	[2,30,31]
Hawkmoth	1600	6100	2.7	26.1	0.298	116°	[2,30,32]

* Approximate estimation using mid-range values, not from the literature.

## Data Availability

Not applicable.
